# Microorganisms in Whole Botanical Fermented Foods Survive Processing and Simulated Digestion to Affect Gut Microbiota Composition

**DOI:** 10.3389/fmicb.2021.759708

**Published:** 2021-12-07

**Authors:** Miin Chan, Di Liu, Yingying Wu, Fan Yang, Kate Howell

**Affiliations:** School of Agriculture and Food, The University of Melbourne, Parkville, VIC, Australia

**Keywords:** water kefir, tibicos, yeasts, lactic acid bacteria, sauerkraut, fermented food, gut microbiota, *in vitro* digestion

## Abstract

Botanical fermented foods have been shown to improve human health, based on the activity of potentially beneficial lactic acid bacteria (LAB) and yeasts and their metabolic outputs. However, few studies have explored the effects of prolonged storage and functional spices on microbial viability of whole fermented foods from fermentation to digestion. Even fewer have assessed their impact on the gut microbiota. Our study investigated the effects of production processes on LAB and yeast microbial viability and gut microbiota composition. We achieved this by using physicochemical assessments and an *in vitro* gastrointestinal and a porcine gut microbiota model. In low-salt sauerkraut, we assessed the effects of salt concentration, starter cultures, and prolonged storage, and in tibicos, prolonged storage and the addition of spices cayenne, ginger, and turmeric. In both food matrices, LAB counts significantly increased (*p*<0.05), reaching a peak of 7–8 log cfu/g, declining to 6–6.5 log cfu/g by day 96. Yeast viability remained at 5–6 log cfu/g in tibicos. Ginger tibicos had significantly increased LAB and yeast viability during fermentation and storage (*p*<0.05). For maximum microbial consumption, tibicos should be consumed within 28days, and sauerkraut, 7weeks. Simulated upper GI digestion of both products resulted in high microbial survival rates of 70–80%. The 82% microbial survival rate of cayenne tibicos was significantly higher than other treatments (*p*<0.05). 16S rRNA sequencing of simulated porcine colonic microbiota showed that both spontaneously fermented sauerkraut and tibicos increase the relative abundance of *Megasphaera* 85-fold. These findings will inform researchers, producers, and consumers about the factors that affect the microbial content of fermented foods, and their potential effects on the gut.

## Introduction

Botanical fermented foods are microbially transformed plant products rich in health-promoting components. These non-dairy plant-based functional foods potentially manipulate the microbiota-gut-metabolism axis (Mäkinen et al., 2016; Gille et al., 2018). As such, there has been an increase in research characterizing their extracted bioactive components (Değirmencioğlu et al., 2016; Septembre-Malaterre et al., 2018), isolated microbial strains, and their metabolites (Yu et al., 2013; London et al., 2014; Lara-Hidalgo et al., 2017; Angelescu et al., 2019; Romero-Luna et al., 2019, 2020). Few studies explore how being part of a whole fermented food matrix affects microbial viability during fermentation, storage, and gastrointestinal (GI) transit (Buriti et al., 2010; Fiorda et al., 2016; Valero-Cases et al., 2017; Yang et al., 2020). Even fewer investigate the effect of whole fermented food consumption on the gut microbiota (Lavefve et al., 2021). The human gut microbiota is an important dietary target, with its central regulatory role in immune function and energy metabolism (Gille et al., 2018). Botanical fermented foods are cheap, easily made, and consumed globally. This makes them excellent candidates for the dietary management of pro-inflammatory noncommunicable diseases, such as type 2 diabetes and metabolic syndrome.

Whole botanical fermented foods contain components that interact with gut bacteria, including microbes and their metabolites, prebiotic fibers, and other bioactive molecules (Marco et al., 2017). Their potential health effects are likely exerted by lactic acid bacteria (LAB) and yeasts, through biotransformation of inherent ingredients, production of microbial metabolites, or transient integration with gut bacteria (Gille et al., 2018). These microbes have been shown to display similar probiotic activity to dairy-based and human-related strains (La Anh, 2015; Tamang et al., 2016). Recent large-scale metagenomic studies suggest that fermented foods contain health-promoting LAB strains that are closely related to those in the gut microbiome and may well be an important source of commensal strains (Pasolli et al., 2020). In order to exert these potential benefits, LAB and yeasts must endure the rigors of the manufacturing process, storage, consumption, and GI transit (Forssten et al., 2011). Microbial survival in fermented foods is enhanced by integration with appropriate food delivery matrices, due to the presence of protective microbe-digestible sugars (Perrin et al., 2000), prebiotics, and complex microbial communities (Su et al., 2007; Hernandez-Hernandez et al., 2012). To survive GI transit, microorganisms must possess intrinsic acid and bile tolerance (Food and Agriculture Organization of the United Nations and World Health Organisation, 2002; Derrien and van Hylckama Vlieg, 2015); food substrates act as acid buffering agents during digestion (Ranadheera et al., 2010). On reaching the gut, transient fermented food-associated microbes may integrate with gut commensals to produce immunomodulatory and anti-inflammatory metabolites (London et al., 2014; Lara-Hidalgo et al., 2017).

To the best of our knowledge, no studies have investigated the *in vitro* survival of LAB and yeasts and their impact on the gut microbiota when administered as a native part of botanical fermented foods. An accepted cost-effective and ethical way to determine microbial viability is the utilization of *in vitro* GI digestion studies to assess strain resistance to simulated gastric and enteric juices (Buriti et al., 2010; Gbassi et al., 2011). *In vitro* colonic fermentation with next-generation sequencing has proven useful for understanding the transformation and gut-level impacts of dietary compounds, including microbes, prebiotics, and polyphenols (Tsitko et al., 2019; Nissen et al., 2020). Two popular traditional botanical fermented foods, sugar-based tibicos and brine-based sauerkraut, contain potentially probiotic LAB, including *Lactiplantibacillus plantarum* (formerly *Lactobacillus plantarum*), *Levilactobacillus brevis* (formerly *Lactobacillus brevis*), and *Lactobacillus delbrueckii* subsp*. Lactis*; (formerly *Lactobacillus lactis*), yeasts (most notably *Saccharomyces* spp.), and bioactive components (Marsh et al., 2013; Laureys and De Vuyst, 2014; Lavefve et al., 2021). *In vitro* tests of *Saccharomyces cerevisiae* strain C41 and *Lacticaseibacillus paracasei* CT12 (formerly *Lactobacillus paracasei*) isolated from tibicos showed similar probiotic activity to established comparable probiotic strains, with good resistance to gastric pH, bile salts, and *in vitro* digestive fluids (Romero-Luna et al., 2017, 2019, 2020). Autochthonous LAB strains, including *L. plantarum*, from spontaneously fermented sauerkraut are known to have probiotic potential, due to their ability to resist a low acid environment, pancreatin, and bile salts (Yu et al., 2013). LAB strains extracted from sauerkraut have been shown to adhere to CaCo-2 cells and exert antibacterial activity against potential pathogens (Beganović et al., 2014). Leech et al. (2020) recently found that the microbial diversity of tibicos and sauerkraut far exceeded that of dairy-based ferments, as well as containing the largest numbers of potential health-promoting gene clusters. As such, tibicos, and sauerkraut were chosen for our study.

Growth and survival of LAB and yeasts is dependent on the microbial terroir during fermentation and storage: microbial species and strains, inherent food substrates, environmental pH, and organic acid concentrations (Laureys et al., 2019). In the commercial sphere, functional spices, such as ginger, cayenne pepper, and turmeric, are often added to fermented products for sensory and purported health purposes; their effect on microbial proliferation and survival in fermented foods has not been adequately investigated. Similarly, low-salt sauerkraut is increasingly popular, but salt concentrations have been shown to affect microbial diversity and load in the finished product (Di Cagno et al., 2013; Septembre-Malaterre et al., 2018). Starter cultures are commonly used for sauerkraut fermentation and have also been shown to affect microbial growth (Pandey and Garg, 2015; Xiong et al., 2016). Assessing these relationships with physicochemical and *in vitro* systems allows us to develop botanical fermented foods with maximal beneficial impact on intestinal bacteria and thus human health.

In this study, we investigate the effects of storage length, fermentation processes (including salt concentration and use of starter cultures), and functional spices cayenne, ginger, and turmeric on the survival of LAB and yeasts in whole naturally fermented tibicos and sauerkraut, and the subsequent impact on gut bacterial relative abundance in a static *in vitro* digestion and porcine colonic fermentation model. Our findings provide valuable insights into microbial viability in botanical fermented foods from production to consumption, as well as contributing to a useful base for further human studies.

## Materials and Methods

### Tibicos and Sauerkraut Production

Cabbages were purchased from a local retailer and sliced finely before placing into fermentation vessels. Fermentation was carried out anaerobically at 22–25°C for 7days, then stored at 4°C for sauerkraut ripening. Three sauerkraut treatments were produced in triplicate: (1) spontaneous fermentation with 0.6% NaCl and cabbage; (2) spontaneous fermentation with 1.5% NaCl and cabbage; and (3) inoculated fermentation with 0.091% (*w*/*w*) starter cultures (labeled as *L. plantarum, Leuconostoc mesenteroides*, and *Pediococcus acidilactici*; Caldwell, Canada), 0.6% NaCl and cabbage. The sauerkraut treatments were fermented at 20–25°C for 7days, then stored at 4°C for a further 88days. During fermentation and storage, brine (10ml) and cabbage (10g) samples were withdrawn aseptically on days 0, 1, 3, 7, 13, 19, 34, 47, and 95 for pH measurements and microbiological tests. Samples were homogenized and extracts collected, then stored at −20°C for further chemical composition determination.

The primary fermentation of tibicos was performed to ensure that the grains were active and fresh for the subsequent fermentation. Tibicos grains were sourced from a local producer. For every 60g of wet-weight of tibicos grains, 1L of 10% (*w*/*v*) sterilized sucrose solution was supplemented with half an organic dried fig. Tibicos mixtures were incubated at 20–25°C for 72h, after which the tibicos were separated from the liquor and recultivated in new sucrose solution under the same conditions. Tibicos from the primary fermentations were evenly divided into sterilized bottles. Four tibicos treatments were prepared in triplicate: (1) plain tibicos without botanic flavoring powder; (2) ginger tibicos with 0.5% (*w*/*v*) organic ginger powder (*Zingiber officinale*); (3) cayenne tibicos with 0.125% (*w*/*v*) organic cayenne powder (*Capsicum frutescens*); and (4) turmeric tibicos with 0.25% (*w*/*v*) organic turmeric powder (*Curcuma longa*). All botanic flavoring powders were purchased from a local retailer. After the 72-h primary fermentation with tibicos grains, flavoring powders were added and the four treatments were anaerobically incubated at 20–25°C for 48h, then stored at 4°C for a further 91days. Samples were aseptically extracted (1ml) every 24h of primary fermentation and on storage days 12, 19, 33, 47, and 96 for pH measurements and microbiological tests. Length of storage was based on the commercial shelf lives of these products.

### Microbial Enumeration

Viable microbial counts were determined using the methods of De Angelis et al. (2015) and Fischer et al. (2014) with slight modifications. To enumerate LAB, yeasts, and molds, serial dilutions of samples were plated onto modified de Man, Rogosa, and Sharpe (MRS) agar (with 4mg/L cycloheximide) and yeast extract peptone dextrose medium (YPD; with 10mg/L chloramphenicol and 100mg/L ampicillin). For some samples, *Lactobacillus* anaerobic MRS agar with vancomycin and bromocresol green (LAMVAB) agar was also used for LAB enumeration. All types of agar plates were incubated in a constant temperature incubator at 28°C for 3days.

### Chemical Analysis

The pH was determined with a Hanna HI 5221 pH meter (Hanna Instrument, Melbourne, Australia). Corresponding enzymatic assay kits from Megazyme, Bray, Ireland^1^ were used to measure lactate (K-LATE) and acetate (K-ACET); sucrose, glucose and fructose (K-SUFRG); ethanol (K-ETOH), mannitol (K-MANOL), and glycerol (K-GCROL); and ascorbic acid content (K-ASCO). All reagents came from the enzymatic kit and determination processes were conducted based on the manufacturer’s instructions with some modifications for the determination of sugars.

### Simulated Upper Gastrointestinal Digestion and Colonic Fermentation

The simulated upper gastrointestinal digestion and colonic fermentation tests were conducted according to a combination of the consensus harmonized static *in vitro* digestion model (Minekus et al., 2014) and the protocol by Sirisena et al. (2018) with slight modifications (below). We used feces from healthy pigs for colonic fermentation. Samples of both sauerkraut and tibicos were taken on day 34 for administration to the *in vitro* digestive system. This time point ensured that there was a high microbial load (around 7–8 log cfu/ml) and considered the typical length of storage in a commercial setting.

#### Digestive Fluids and Fecal Slurry

Simulated saliva fluid (SSF), simulated gastric fluid (SGF), simulated intestinal fluid (SIF), enzyme (amylase, porcine pepsin, pancreatin, and bile) solution, 0.1M phosphate buffer, basal media, and fecal slurry were prepared according to the methods prescribed by Sirisena et al. (2018). All digestive fluids, enzyme solutions, and fecal slurries were utilized within 1h of preparation.

#### *In vitro* Digestion Simulation

##### Oral Digestion

The oral stock solution consisted of 1.75ml of SSF solution, 0.25ml fresh salivary α-amylase solution of 1,500U/ml (dilute the original solution with SSF solution), 12.5μl of 0.3mol/l CaCl_2_(H_2_O)_2_, and 487.5μl of water. To this mixture, 1ml of sauerkraut brine and 1.5g finely minced sauerkraut, or 2.5ml of tibicos was added to achieve the final ratio of sample: stock solution=1:1. All tubes were tightly sealed, then mixed by a pre-warmed 37°C shaking incubator with a constant parameter of 200rpm for 2min. An oral bolus was obtained.

##### Gastric Digestion

The 5ml oral bolus was mixed with 3.75ml of SGF solution, 0.8ml fresh porcine pepsin stock solution of 25,000U/ml (dissolved in SGF solution), 2.5μl of 0.3mol/l CaCl_2_(H_2_O)_2_, and 387.5μl of Milli-Q water. The final sample-to-stock ratio was 1:1. The mixture pH was adjusted to 3.0 *via* the addition of required HCl. All tubes then underwent shaking for 2h at 37°C. Gastric chyme was obtained.

##### Small Intestinal Digestion

The gastric chyme was mixed with 5.5ml SIF solution, 2.5ml of fresh pancreatin solution of 800U/ml (pre-warmed to 37°C and well-shaken), 1.25ml fresh fed state bile (49mg/ml), 20μl of 0.3mol/L CaCl_2_(H_2_O)_2_, and 655μl (sauerkraut) or 1.152ml (tibicos) of Milli-Q water. The pH of the mixture was adjusted to 7.0 *via* addition of 1mol/l of NaOH, before 2h shaking incubation at 37°C. For sauerkraut samples, the small intestinal digesta was centrifuged at 5,000rpm for 15min. 1ml of sauerkraut small intestinal digesta was collected for microbiological determination. As the tibicos sample was a clear liquid containing minimal solids, 1ml of the small intestinal digesta was directly sampled and stored, rather than being centrifuged to acquire precipitate fractions. Precipitate fractions were separated and stored at 4°C for colonic fermentation.

#### Microbial Enumeration

Triplicate samples of all treatments prior to and following gastric and small intestinal digestion were serially diluted. One hundred microliter of each dilution was spread-plated on MRS, YPD, and LAMVAB agar for aerobic and anaerobic microbial enumeration. Triplicate blanks – fecal samples without small intestinal digesta – were also diluted to enumerate the original existing LAB and yeast in porcine feces. The plates were then incubated.

#### Colonic Fermentation

Here, 1ml tibicos small intestinal digesta or 1ml sauerkraut precipitate fraction was mixed with 5ml each of pre-warmed basal medium and fecal slurry (Sirisena et al., 2018). All tubes were flushed with nitrogen gas for several seconds to remove air. This was followed by a 24h fermentation at 37°C in the anaerobic chamber of a shaking incubator. An aliquot of 1ml from each resulting liquid solution was taken for microbiological tests, while the remainder was frozen at −20°C for DNA extraction and sequencing.

### DNA Extraction and Sequencing

Genomic DNA was extracted from the duplicated colonic fermentation samples, using PowerSoil™ DNA Isolation kits (QIAgen, CA, United States), as per the kit protocol. A blank (fecal slurry with digestive fluids and no fermented food) and a control (colonic digesta) were included. DNA extracts were submitted to the Australian Genome Research Facility (AGRF) for amplification and sequencing. To assess the bacterial communities, 16S rRNA gene V3–V4 region was amplified using the universal primer pairs 341F/806R (Yu et al., 2005), followed by 300bp paired-end sequencing on an Illumina MiSeq (San Diego, CA, United States). Raw sequences were processed using QIIME v1.9.2 (Caporaso et al., 2010). Operational taxonomic units (OTUs) were assigned using UCLUST open-reference OTU-picking workflow with a threshold of 97% pairwise identity (Edgar, 2010). Taxonomy was assigned to OTUs in QIIME using the Ribosomal Database Project (RDP) classifier (Wang et al., 2007) against the GreenGenes bacterial 16S rRNA database (v13.8; DeSantis et al., 2006).

### Data and Statistical Analysis

All experiments were performed in triplicate, with results reported as the mean±standard deviation. Differences among samples were assessed using one-way ANOVA and Fisher pairwise comparisons at a significance level of 0.05 using Minitab18.

## Results

Our study evaluated microbial viability of LAB and yeasts in sauerkraut and tibicos during fermentation and storage, using a plate colony-counting method. pH value and chemical composition changes were tested using appropriate methods. Static *in vitro* digestion tests were performed to determine microbial survival in a simulated gastric and small intestinal model. Finally, colonic fermentation was used to determine the effect of LAB and yeast on porcine gut bacteria.

### pH and Chemical Composition

For all tibicos and sauerkraut treatments, we measured organic acid (lactic acid, acetic acid, and ascorbic acid), alcohol (ethanol, glycerol, and mannitol), and sugar (fructose, glucose, and sucrose) content, and pH, during fermentation and storage (Supplementary Material). During early fermentation, the pH of both sauerkraut and tibicos dropped below 3.5. In tibicos, lactic and acetic acid levels increased, as did ethanol, glycerol, and mannitol levels, while all carbohydrate levels fell. In sauerkraut, lactic and acetic acid levels increased, as did ethanol and mannitol levels, while ascorbic acid levels dropped. During tibicos storage, pH trended downward, falling to around 3. Lactic and acetic acid levels dropped slightly, then gradually increased and plateaued at day 50, with only ginger tibicos experiencing a continuing upward trend. Ethanol, glycerol and mannitol levels dipped then increased and plateaued at day 50. During sauerkraut storage, pH remained stable. Lactic and acetic levels increased, except for the inoculated sauerkraut which showed a precipitous drop in acetic acid from day 34 onward. Ascorbic acid levels of all sauerkraut continued to decline. Ethanol levels increased, except in inoculated sauerkraut where it plateaued; mannitol levels in all sauerkraut plateaued. Significant differences between the treatments were not found (*p*>0.05).

### Microbial Growth and Survival During Fermentation and Storage

**Figure 1:**
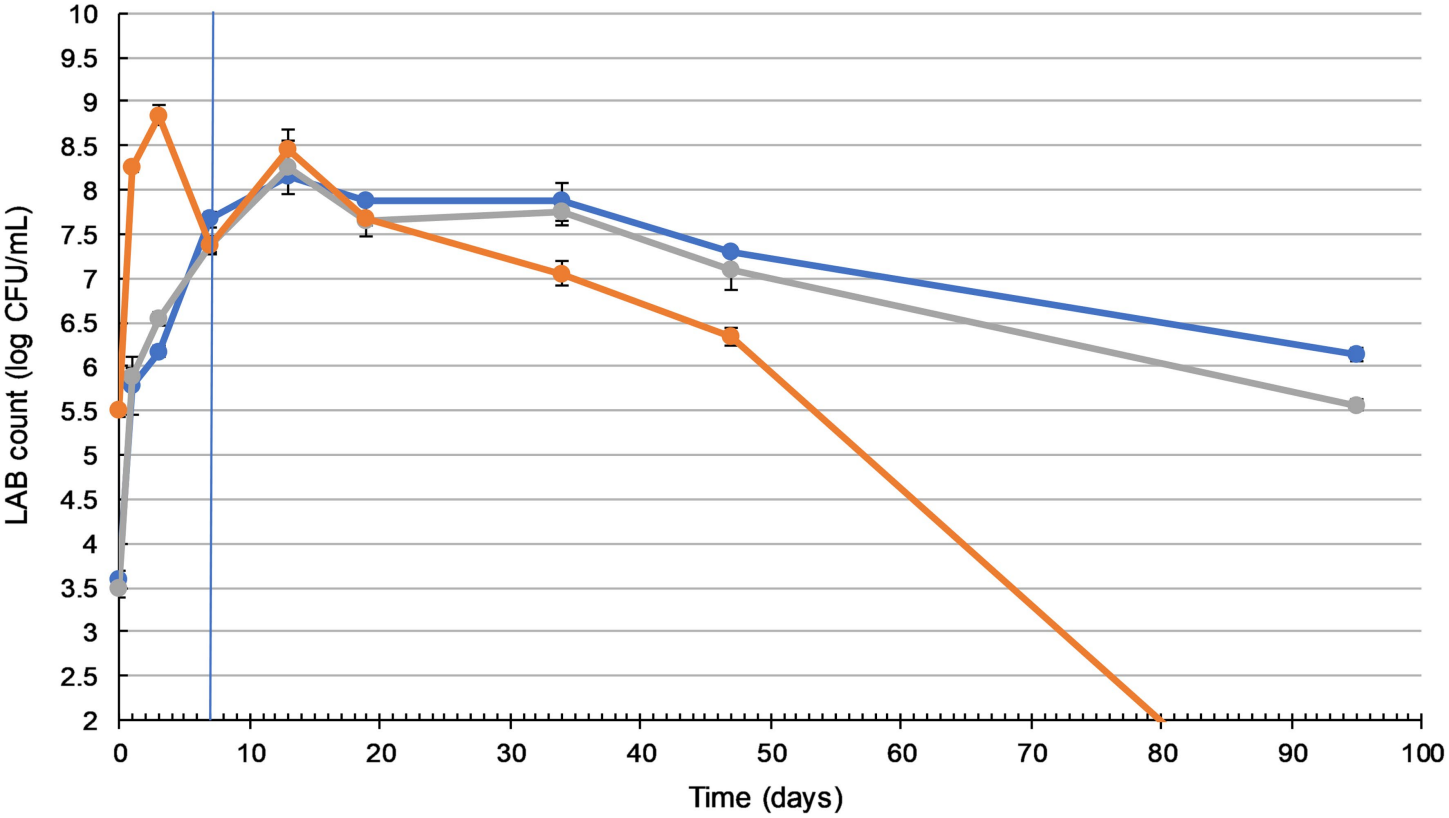
Lactic acid bacteria (LAB) counts in sauerkraut remain sufficiently high during storage. Changes in LAB count during fermentation (20–25°C for 7days) and storage (4°C for a further 88days) of three sauerkraut treatments. (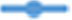) spontaneous fermentation with 0.6% salt; (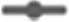) spontaneous fermentation with 1.5% salt; (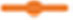) inoculated fermentation with 0.91% (*w*/*w*) starter cultures and 0.6% salt. Sampled on days 0, 1, 3, and 7 (during fermentation), and on days 13, 19, 34, 47, and 95. The timeline of fermentation and storage is separated by the vertical blue line. The results were expressed as mean standard deviation (*n*=3) for each treatment group.

Our study investigated LAB and yeast survival during fermentation and storage. During the 7-day fermentation and a further 88days of storage, samples were taken, diluted, and plated for microbial enumeration (Figure 1).

Sauerkraut made with different salt concentrations did not show any significant difference in LAB counts (*p*>0.05). The inoculation of starter culture increased initial LAB counts by more than 2 log units, from 3.5 to 5.5 log cfu/ml. Inoculated and spontaneously fermented sauerkraut then followed a similar LAB count trend, with a significant gradual increase (*p*<0.05) to a peak of around 8 log cfu/ml on day three (inoculated), during fermentation, and day 13 (spontaneous), during storage. Inoculated sauerkraut LAB counts dropped to undetectable levels by the end of storage, while spontaneously fermented sauerkraut LAB counts remained stable at around 6–7 log cfu/ml from day 47 to 95.

Yeast enumeration was also carried out (Supplementary Material), with a peak of around 5.6 log cfu/ml and no significant difference between the treatments (*p*>0.05). By the 3rd day of fermentation, no yeast or other molds were detected in any of the sauerkraut samples.

**Figure 2:**
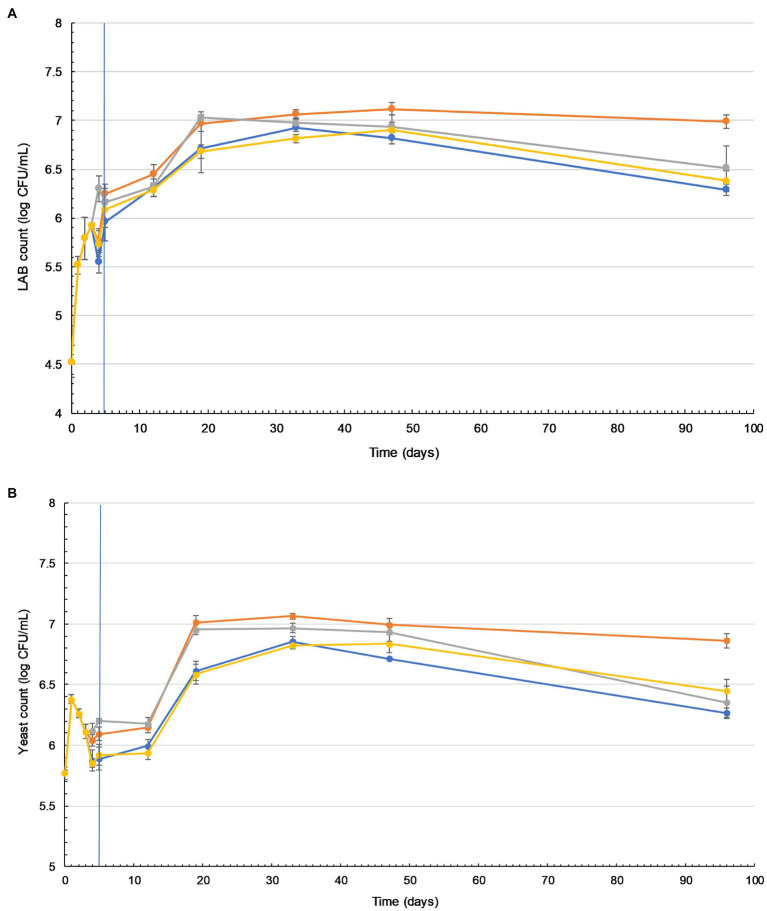
Lactic acid bacteria (LAB) and yeast counts in tibicos increase during storage and are significantly higher when ginger is added. Changes in viable LAB **(A)** and yeast **(B)** counts in four tibicos treatments during fermentation (20–25°C for 3days with tibicos; and with three different additives added on day 3 for 2days of secondary fermentation without tibicos and storage at 4°C for a further 86days). (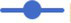) Plain tibicos; (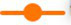) tibicos with 0.5% (*w*/*v*) organic ginger powder; (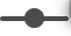) tibicos with 0.125% (*w*/*v*) organic cayenne powder; (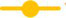) tibicos with 0.25% (*w*/*v*) organic turmeric powder. Sampled daily during fermentation and on days 12, 19, 33, 47, and 96. The timeline of fermentation with the tibicos grains and separation to enter storage is indicated by the vertical blue line. The results were expressed as mean standard deviation (*n*=3) for each treatment group.

The LAB count in all tibicos treatments significantly increased during fermentation (*p*<0.05), with slight fluctuations on addition of functional spices (Figure 2A). There was no significant difference between the treatments after 5days of fermentation (*p*>0.05). During storage, the LAB count of all treatments increased, reaching their peak of around 7 log cfu/ml on day 19 for cayenne, day 35 for plain and day 47 for ginger and turmeric tibicos. The LAB count of ginger tibicos remained stable and was significantly higher at 7 log cfu/ml than the other three treatments on day 96 (*p*<0.05). The LAB count of the other three treatments gradually decreased to around 6–6.5 log cfu/ml. Overall, LAB content in tibicos increased 60-fold over 96days (Figure 2A).

Yeast counts increased slightly during the initial fermentation, peaking at around 6 log cfu/ml (Figure 2B). During secondary fermentation, the yeast counts of the ginger and cayenne tibicos did not differ from each other but were significantly higher than the plain and turmeric tibicos (*p*<0.05). During early storage, the yeast counts of all four tibicos treatments experienced a plateau, followed by a significant surge to their highest points of around 7 log cfu/ml, followed by a slow decline over time. In the last month of storage, yeast counts of plain, cayenne, and turmeric tibicos dropped significantly to around 6 log cfu/ml. Ginger tibicos yeast counts remained stable at approximately 7 log cfu/ml. On day 96, ginger tibicos had significantly higher yeast counts than the other three treatments (*p*<0.05; Figure 2B).

### Survival of LAB and Yeasts Under Simulated GI Conditions

Microbial enumeration showed that LAB in sauerkraut and tibicos were able to survive fermentation and periods of storage in sufficient probiotic numbers. Our study then investigated if these microbes could tolerate low pH, bile salts, pepsin, and pancreatin in a simulated gastric and small intestinal model.

**Figure 3:**
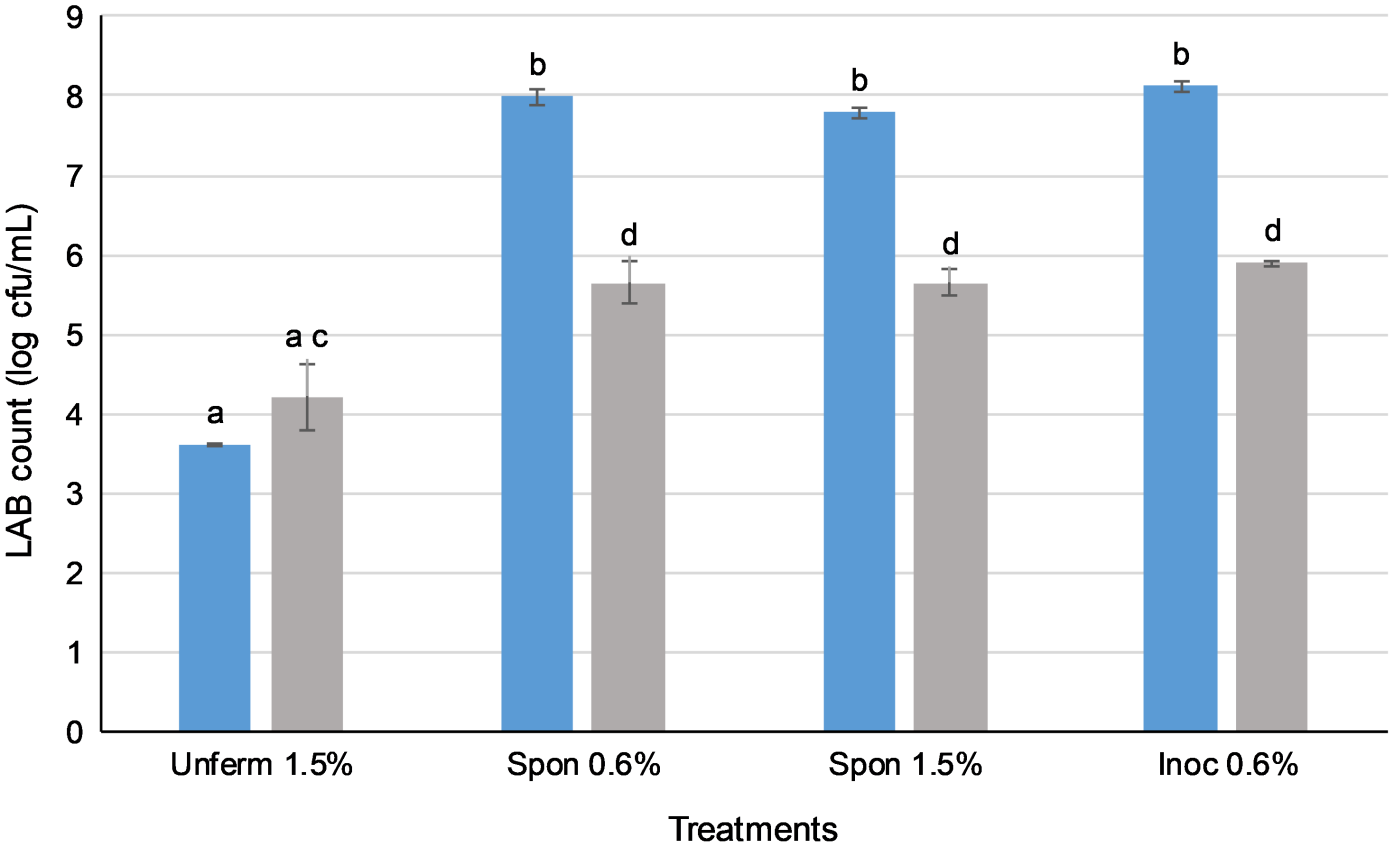
Lactic acid bacteria in sauerkraut survive simulated oral, gastric, and small intestinal conditions. (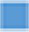) Total LAB plate counts before *in vitro* digestion; (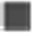) total LAB plate counts after simulated GI conditions. The results were expressed as mean standard deviation (*n*=3) for each treatment group. Means with the same letter are not significantly different (*p*<0.05).

Prior to *in vitro* digestion, there were no significant differences in LAB counts between sauerkraut treatments (Figure 3), all of which were around 8 log cfu/ml. The LAB count of the unfermented cabbage and salt mixture was around 3 log cfu/ml. After the small intestinal phase, LAB counts of all the sauerkraut treatments were significantly lower than prior to GI simulation (*p*<0.05), dropping to around 6 log cfu/ml. The LAB count of the unfermented cabbage treatment increased, but not significantly. There were no significant differences between the sauerkraut treatments, though their LAB counts were all significantly higher than the unfermented cabbage (*p*<0.05). The survival rate of LAB was not significantly different between sauerkraut treatments at around 72% (Table 1), but the unfermented cabbage treatment had a significantly higher survival rate of LAB than the sauerkraut treatments (*p*<0.05).

**Table 1:** Salt concentration and inoculation do not affect lactic acid bacteria (LAB) survival rate during *in vitro* digestion.

Treatments	LAB survival rate (%)
Unfermented cabbage+1.5% salt	117.1±0.12.3^a^
Spontaneously fermented sauerkraut+0.6% salt	71.7±2.3^b^
Spontaneously fermented sauerkraut+1.5% salt	72.5±1.7^b^
Inoculated sauerkraut+0.6% salt	72.7±0.5^b^

*Percentage survival of sauerkraut-associated LAB after simulated gastric and small intestinal digestion. Means within a column with different superscripts (a, b) are significantly different (p<0.05). The results expressed as mean (n=3)±SD (standard deviation)*.

**Figure 4:**
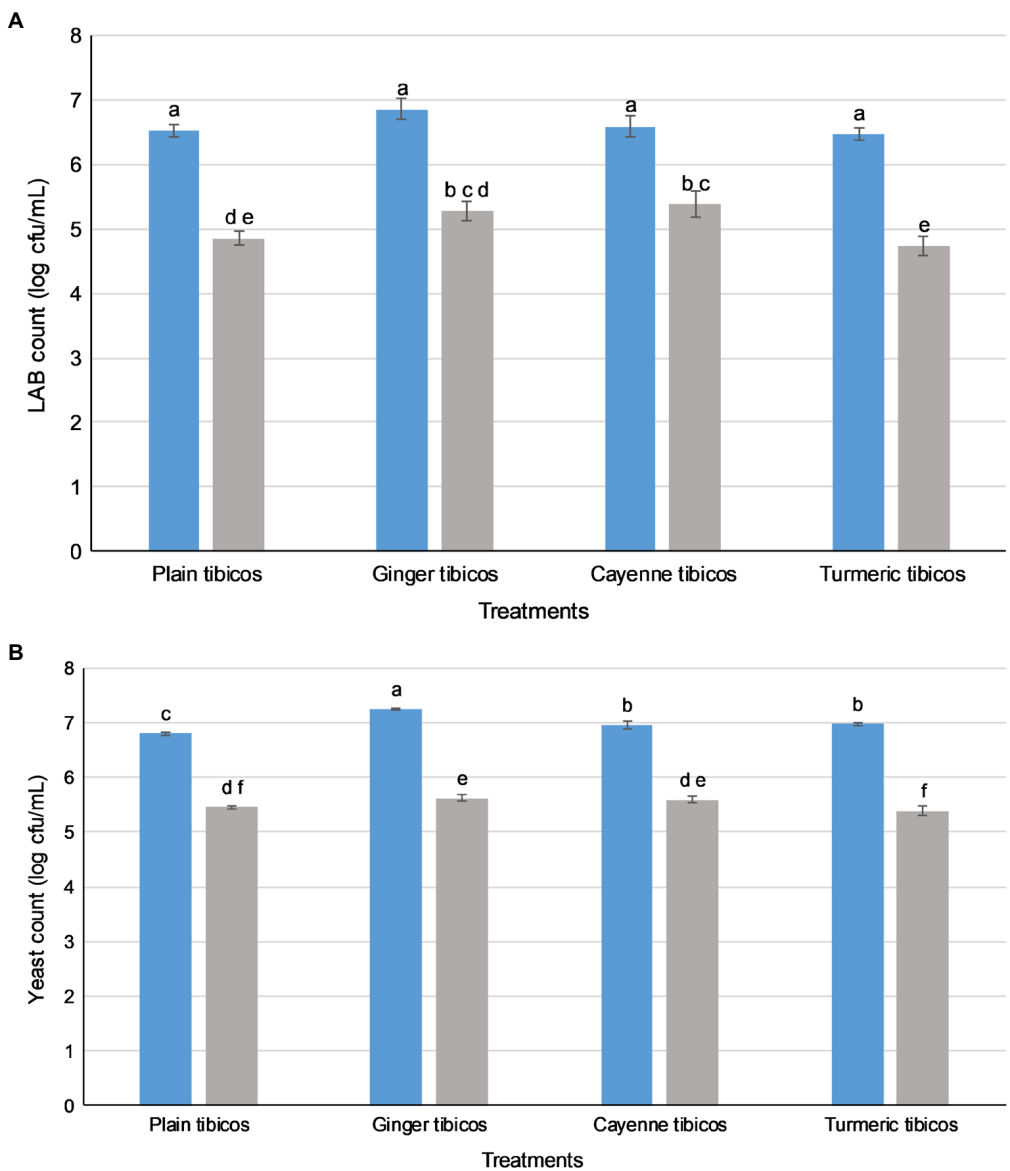
Lactic acid bacteria (LAB) and yeasts in tibicos survive simulated oral, gastric, and small intestinal conditions. LAB (A) and yeast (B) counts. (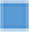) LAB or yeast counts before in vitro digestion; (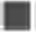) LAB or yeast counts after simulated GI conditions. The results were expressed as mean standard deviation (n=3) for each treatment group. Means with the same letter are not significantly different (p<0.05).

In all four tibicos treatments, both LAB and yeast counts fell significantly after the small intestinal phase (*p*<0.05; Figure 4). LAB counts experienced an approximate 2 log loss to 5 log cfu/ml, while yeast counts dropped by 1 log to 6 log cfu/ml. Cayenne tibicos had a significantly higher LAB count compared to plain and turmeric tibicos (*p*<0.05), and ginger tibicos had a significantly higher LAB count than turmeric tibicos (*p*<0.05). Ginger tibicos had significantly higher yeast count than both plain and turmeric tibicos, while cayenne tibicos had significantly higher yeast counts than turmeric tibicos. The addition of cayenne powder to tibicos significantly improved the survival rate of LAB during simulated gastric and small intestinal digestion (*p*<0.05), compared to ginger and turmeric. Ginger tibicos had a significantly higher rate of LAB survival than turmeric tibicos (*p*<0.05), but neither treatment had significantly higher LAB survival rates than plain tibicos (*p*<0.05). Yeast survival rates in ginger and turmeric tibicos were significantly lower than plain and cayenne tibicos (*p*<0.05).

### Impact of Digested LAB and Yeasts on Gut Microbiota

LAB and yeasts from the fermented foods were able to survive simulated digestion in high numbers, but we wanted to know if these microbes could affect the complex microbial community of the large intestine. To observe these effects, after *in vitro* gastric and small intestinal digestion, all tibicos and sauerkraut samples then underwent 24-h colonic fermentation using porcine feces. Bacterial communities were profiled by extracting DNA from all samples and performing 16S rRNA amplicon sequence analysis. A total of 861,164 16S rRNA high-quality sequences were generated from all the samples, which were clustered into 1,891 bacterial OTUs with a threshold of 97% pairwise identity.

#### Microbial Enumeration

The fecal slurry contained 11 log cfu/ml LAB and 10.5 log cfu/ml yeast. All tibicos and sauerkraut small intestinal digesta were mixed with the fecal slurry before colonic fermentation. After colonic fermentation, the control contained 8 log cfu/ml LAB and 5.6 log cfu/ml yeast. Tibicos samples contained 7–8 log cfu/ml LAB and 6 log cfu/ml yeast; sauerkraut samples had 8 log cfu/ml LAB and 5–6 log cfu/ml yeast remaining (Supplementary Material).

#### Taxonomic Composition of Blank and Control Samples

**Figure 5:**
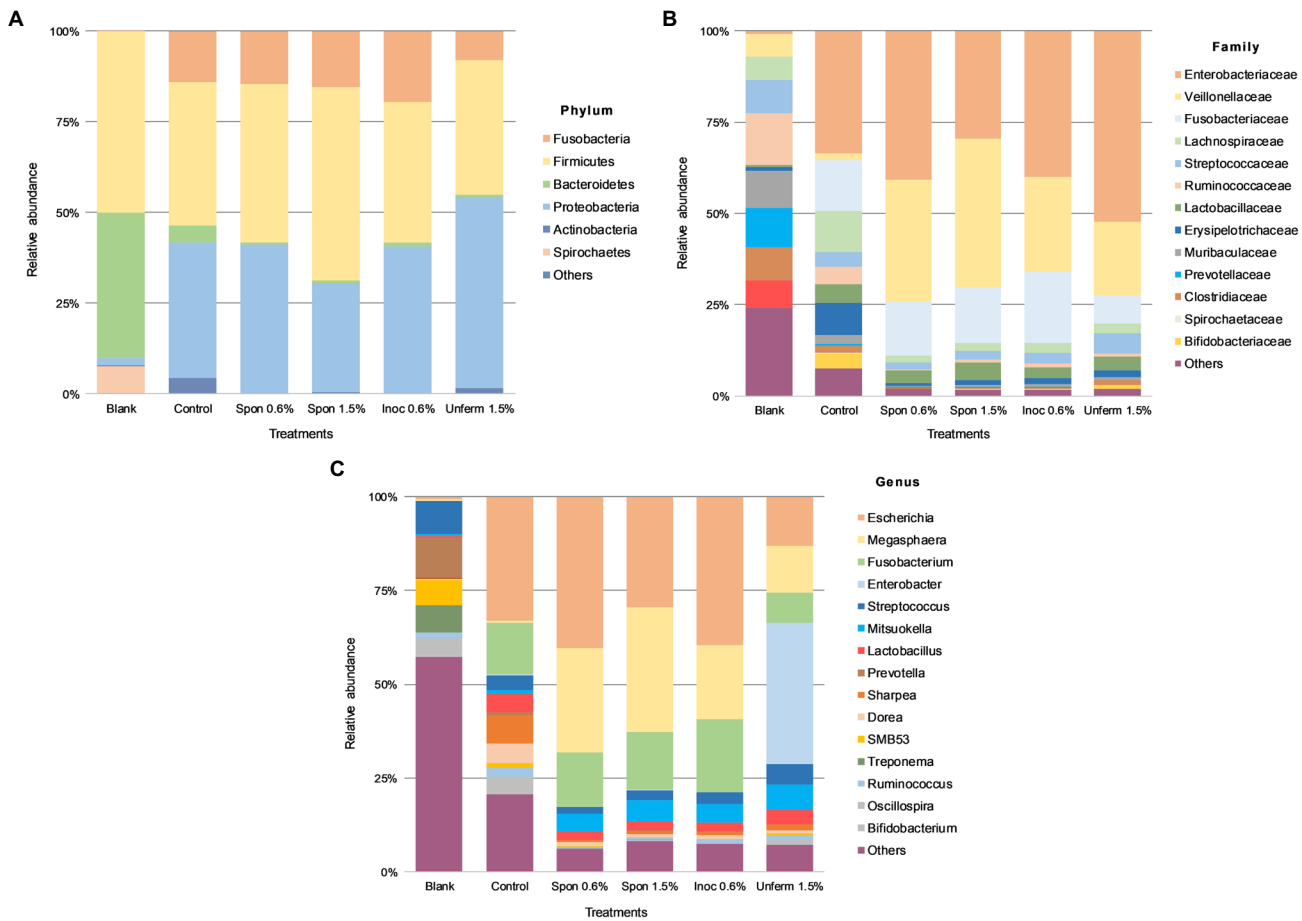
Colonic fermentation of digested sauerkraut affects the final gut bacterial profile. Microbial taxa are characterized to the phylum **(A)**; family **(B)**; genus **(C)** levels. Dominant bacterial taxa are given with greater than 1.0% relative abundance. Blank: fecal slurry with digestive fluids. Control: fecal slurry with digestive fluids post-colonic fermentation. Spon 0.5%: spontaneously fermented sauerkraut with 0.6% salt; Spon 1.5%: spontaneously fermented sauerkraut with 1.5% salt; Inoc 0.6%: inoculated sauerkraut with 0.6% salt; Unferm 1.5%: unfermented cabbage with 1.5% salt.

**Figure 6:**
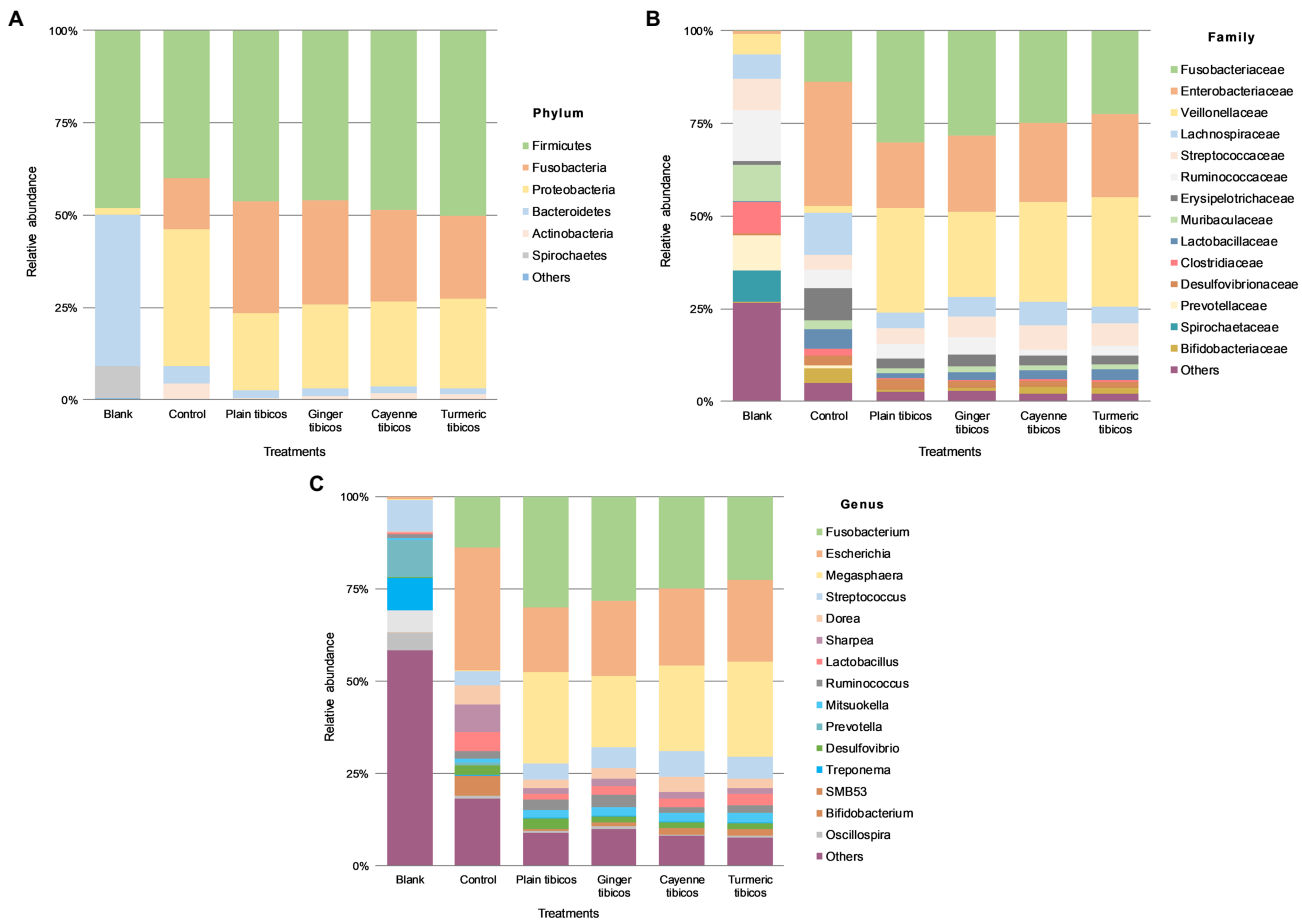
Colonic fermentation of digested tibicos affects the final gut bacterial profile. Microbial community structures in the four tibicos treatments. Microbial taxa are characterized to the phylum **(A)**; family **(B)**; genus **(C)** levels. Dominant bacterial taxa are with greater than 1.0% relative abundance. Blank: fecal slurry with digestive fluids. Control: fecal slurry with digestive fluids post-colonic fermentation.

In the blank fecal samples, Bacteroidetes, Firmicutes, and Spirochetes made up >97% of the phyla detected (Figures 5, 6). The relative abundance of the phylum Firmicutes was higher than Bacteroidetes (Figures 5A, 6A). At the family level (Figures 5B, 6B), *Ruminococcaceae* had the highest relative abundance at around 14%, with *Muribaculaceae*, *Prevotellaceae*, *Spirochaetaceae*, and *Clostridiaceae* at between 8 and 10% each. At the genus level, *Prevotella* dominated at 11%, with *Streptococcus* relative abundance (Figures 5C, 6C) at 9%. *Lactobacillus* relative abundance was at 0.4% and *Bifidobacterium* at 0.07%, while *Enterobacter* had 0% relative abundance.

After colonic fermentation, Firmicutes, Proteobacteria, and Fusobacteria comprised >91% of the phyla detected in the control, with relative abundance of Bacteroidetes dropping from 40 to 5% (Figures 5A, 6A). The relative abundance of Firmicutes was much higher than Bacteroidetes after simulated colonic digestion. At the family level, in comparison with the blank, *Enterobacteriaceae* (0.7% vs. 34%) (Figures 5B, 6B), *Fusobacteriaceae* (0.01% vs. 14%), and *Lachnospiraceae* (6 vs. 11%) dominated. *Lactobacillaceae* increased tenfold from 0.4 to 5%. At the genus level (Figures 5C, 6C), compared to the blank, the dominant genera were *Escherichia* (0.7–33%), *Fusobacterium* (0.01–14%), and *Sharpea* (0.02–8%). *Lactobacillus* relative abundance increased from 0.4 to 5%. The relative abundance of several families decreased: *Clostridium* (3% to 0.5), *Streptococcus* (9–4%), *Prevotella* (11–0.6%), and *Treponema* (7–0.08%).

#### Taxonomic Composition of Sauerkraut and Tibicos Colonic Digesta

Relative abundance across all sauerkraut treatments was at <1% on phylum and family levels; at genus level, relative abundance was at <0.1% across all treatments (Figure 5). After colonic digestion, Firmicutes, Proteobacteria, and Fusobacteria comprised >98% of phyla detected in the unfermented cabbage and all sauerkraut treatments. These phyla also dominated in the control sample. Compared to the control at 5%, Bacteroidetes relative abundance dropped to between 0.3 and 1%; Firmicutes relative abundance in the control was at 40% compared to the treatments which were between 37 and 53%. At the family level (Figure 5B), like the control, *Enterobacteriaceae* had the highest relative abundance in all sauerkraut treatments (between 40 and 52%); *Veillonellaceae* expanded from 1.8% in the control, to between 20 and 34% in sauerkraut samples. The exception was spontaneously fermented sauerkraut with 1.5% salt, where *Veillonellaceae* was at 40% relative abundance and *Enterobacteriaceae* at 30%. This also differed from the control where *Enterobacteriaceae*, *Fusobacteriaceae* and *Lachnospiraceae* dominated. Like the control, *Lactobacillaceae* relative abundance increased compared to the blank. At the genus level (Figure 5C), like the control, *Escherichia* was most abundant. Compared to the control (0.4%), *Megasphaera* relative abundance increased around 85-fold in the spontaneously fermented sauerkraut (28 and 33%) and to 20% in the inoculated sauerkraut and 13% in the unfermented cabbage. *Fusobacterium* was also relatively dominant at 15–20% in the sauerkraut samples and 8% in the unfermented cabbage. In the unfermented cabbage, *Enterobacter* dominated at 38%, in contrast to the control and sauerkraut treatments. Compared to the control at 5%, sauerkraut *Lactobacillus* relative abundance was halved, while *Bifidobacterium* relative abundance dropped from 4% to around 0.2%. In the unfermented cabbage treatment, there was less of a reduction compared to control, with 4% *Lactobacillus* and 1% *Bifidobacterium* relative abundance.

Relative abundance across all tibicos treatments was at <1% on phylum, genus, and family levels (Figure 6). Like the control colonic digesta, Firmicutes, Fusobacteria, and Proteobacteria comprised >97% of phyla detected in the tibicos treatments after simulated colonic fermentation (Figure 6A). There was no difference between the treatments. Bacteroidetes relative abundance was around 2%, compared to 5% in the control, while Firmicutes relative abundance remained the same as the blank and control. At the family level (Figure 6B), *Fusobacteriaceae* (22–30%), *Enterobacteriaceae* (18–22%), and *Veillonellaceae* (22–30%) dominated in all four tibicos treatments. This differed from the control, where *Lachnospiraceae* was more abundant than *Veillonellaceae*. When taxonomic composition was examined at genus level (Figure 6C), the dominant genus in all tibicos treatments was *Fusobacterium* at between 22 and 30% relative abundance, twice as abundant as in the control. *Escherichia* relative abundance halved compared to the control, to around 20%. There was no difference in relative abundance between the tibicos treatments. Compared to the control (0.4%), compression of relative abundance with enrichment in *Megasphaera* (between 19 and 25%) was observed. In the control, *Lactobacillus* relative abundance was approximately 5%; in plain tibicos, it was reduced to 1.4%, while the spice-added tibicos were between 2 and 3%. Similarly, *Bifidobacterium* relative abundance was 4% in the control, while plain tibicos was reduced tenfold at 0.4%; the spice-added treatments were between 0.8 and 1.6%.

## Discussion

Botanical fermented food components are of increasing interest as potential beneficial modulators of gut microbiota for the management of metabolic noncommunicable diseases (Şanlier et al., 2019), but few studies focus on their application as whole foods from production to digestion. Our study showed that LAB and yeasts in sauerkraut and tibicos can be manipulated during production and storage for maximal microbial viability, may have higher survival rates when digested in their native whole food matrix, and can impact the relative abundance of gut bacteria.

### Storage Length and Starter Cultures Affect Microbial Viability of LAB and Yeasts

The factors that impact LAB and yeast viability in both tibicos and sauerkraut are well studied. These include ingredient variation, salt concentration, use of starter cultures, and storage conditions, which affect metabolite concentration, pH and oxygen levels. A comprehensive review of tibicos microbial dynamics during production and storage has been performed by Lynch et al. (2021), Laureys and De Vuyst (2014), Laureys et al. (2017, 2018, 2019, 2021), and for sauerkraut, this area has been recently reviewed by Zabat et al. (2018) and Yang et al. (2020).

In our work, the initial LAB population on the surface of shredded cabbage was around 3 log cfu/ml, which is within the typical range of fresh cabbage microflora (Fleming et al., 1988). As shown in Figure 1, inoculation with a starter culture labeled as containing *L. plantarum*, *L. mesenteroides*, and *P. acidilactici* caused an expected initial 2 log increase in the LAB count. For both spontaneously fermented and inoculated sauerkraut, LAB counts peaked at 8–8.5 log cfu/ml on day 13. A similar result was observed in Lu et al.’s (2003) study where LAB counts peaked at 8.4 log cfu/ml on day 15. However, Beganović et al. (2011) reported that inoculated sauerkraut required 21days of fermentation to reach a peak of 7.63 log cfu/ml, and 28days for spontaneously fermented sauerkraut (6.26 log cfu/ml). These differences may be attributed to higher 4.0% NaCl concentration, which inhibits the utilization of sucrose, reduces LAB metabolic rates, and leads to sauerkraut maturation delays (Xiong et al., 2016). The LAB counts of the spontaneously fermented sauerkrauts dropped to between 6 and 7 log cfu/ml by day 95. This is typically seen in sauerkraut fermentations, due to the inhibitory effect of lactic acid and the gradual depletion of sugars (Xiong et al., 2012). In contrast, by day 95, inoculated sauerkraut LAB counts had dropped to undetectable levels. These results differ from those of Pandey and Garg (2015), who found that the LAB count of inoculated sauerkraut was consistently higher than that of spontaneously fermented sauerkraut. Conversely, Xiong et al. (2016) found that the desired rapid accumulation of lactic acid and subsequent low pH environment of inoculated sauerkraut may restrict LAB growth, leading to faster reduction in microbial proliferation. Commercial sauerkrauts are often fermented for 4–6weeks, with a shelf life of up to 12months. Our study established that, in order to maximize its probiotic potential and meet the requirements for probiotic foods (>6 log cfu/ml), sauerkraut should be consumed within 7weeks of manufacture.

In our study of tibicos, we were interested in understanding the effects of prolonged storage (96days), as this is a typical shelf life of commercial tibicos. We were unable to find other studies of tibicos where storage exceeded 35days. Our study observed the same trends as other tibicos studies (Miguel et al., 2011; Laureys and De Vuyst, 2014; Viana et al., 2017) in pH, metabolite production and carbohydrate depletion (Supplementary Material), and microbial counts during fermentation (Figure 2). Peak microbial counts during fermentation did differ between studies; this is likely due to tibicos grains from diverse geographic locations and the varied array of substrates used. In our study and those mentioned above, there was initial LAB growth with yeast suppression during initial fermentation. This was followed by an increase in yeast populations over 21days, as LAB growth slowed but remained steady. Yeast counts in all treatments peaked at around 7 log cfu/ml on day 35. LAB counts of all treatments increased, reaching a peak of around 7 log cfu/ml on different days: cayenne on day 19, plain on day 35, and ginger and turmeric on day 47. These differences are likely due to the varying availability of spice-related nutrients during tibicos fermentation, which impact substrate consumption, metabolite concentrations, and microbial growth and species diversity (Laureys and de Vuyst, 2017; Laureys et al., 2018). Then followed a gradual decline in the viable counts of both yeasts and LAB, with all treatments except ginger tibicos at 6–6.5 log cfu/ml by day 96. A 2016 study by Fiorda et al. (2016) on honey must kefir showed similar results, with yeast and LAB counts of 6–7 log cfu/ml remaining stable until the end of their 35-day storage period. In our study, the LAB and yeast counts in ginger tibicos remained stable and were significantly higher than the other three treatments on day 96 (*p*<0.05); this is discussed further below. Overall, we found that in order to ensure maximum total microbial counts at consumption, tibicos should be stored for around 28days at 4°C, but no longer.

### The Addition of Ginger Significantly Increased and Sustained Microbial Viability of LAB and Yeasts in Tibicos During Fermentation and Storage

Spices are often used as functional ingredients and bio-preservatives in fermented foods, prized for their nutrient content, high antioxidant capacity, and aromatic flavor properties (Wahba et al., 2009). Ginger, cayenne, and turmeric possess anti-inflammatory properties, known to be beneficial for human health (Kunnumakkara et al., 2018). In our study, these spices were added on day 3 for a 48-h secondary fermentation. The pH of the tibicos immediately increased, with ginger and turmeric tibicos pH remaining higher than plain and cayenne tibicos throughout storage (Supplementary Material); microbial counts continued upward following addition of spices (Figure 2). This is likely due to several factors that support microbial metabolism, such as the presence of fiber as a major substrate during secondary fermentation; bioavailable polyphenols; and spice-related micronutrients, such as iron, copper, and amino acids. Çevik et al. (2019) showed that tibicos grains cultivated in honey or grape molasses solutions, both rich in vitamins and minerals, led to a significantly larger increase in LAB and yeast counts by the end of fermentation, compared to sucrose solution. However, in our study, by the end of secondary fermentation on day 5, there was no significant difference between the pH and microbial counts of the four tibicos treatments.

Besides its medicinal uses, ginger (*Z. officinale*) is often employed as a bio-preservative in the food industry due to its antifungal, antimicrobial, and antioxidant properties (Obi, 2015; Arekemase and Babashola, 2019). In the present study, only the addition of ginger significantly increased and sustained both LAB and yeast loads at approximately 7 log cfu/ml (*p*<0.05) during prolonged storage, compared to plain, cayenne, and turmeric tibicos, whose microbial loads declined during the last month of storage (Figure 2). The accelerated growth of LAB in ginger tibicos in our study is consistent with Wang et al. (2017), whose study demonstrated that the addition of ginger extract to pickle fermentation increased LAB growth. However, other studies showed varying results, likely due to the application of different concentrations of ginger in a variety of substrates. Goharjoo et al. (2020) investigated the effect of different concentrations of ginger on LAB counts in fermented carrot juice and found that growth of LAB in a 4% treatment was significantly higher while the pH was lower than in 0 and 8% treatments (*p*<0.01). This is likely due to the antibacterial nature of gingerols (Mahady et al., 2003; Rahmani et al., 2014). Our study observed that despite ginger tibicos having the highest levels of LAB, yeasts, and lactic acid, it reached the highest pH of 3.7. Laureys et al. (2018) showed that in a high nutrient environment, tibicos fermentation proceeds at a faster rate, resulting in high metabolite concentrations without a drop in pH; this protects microbial growth from acidic stress. The pH of ginger tibicos then remained at around 3.3 throughout storage, while the pH of plain, cayenne, and turmeric tibicos fell below 3.3 (Supplementary Material). Silva et al. (2009) showed that when the pH of tibicos falls under 3.3, microbial activity is restricted due to acidity pressure. The growth and maintenance of yeast in concert with LAB concurs with findings of Stadie et al. (2013) regarding their mutualistic relationship. Laureys et al. (2018) showed that nutrient concentration during fermentation is associated with shifts in dominant microbial species, with high nutrient concentrations favoring *L. nagelii* and *S. cerevisiae*.

We note here that in our study, due to consumer-based flavor considerations, spice concentrations varied. Although ginger, at the highest concentration of 0.5%, showed significantly higher LAB and yeast growth, turmeric (0.25%) had a lower microbial load than cayenne (0.125%), which was not significantly different to plain tibicos. The complexity of whole food ingredients is such that equal concentrations of different spices do not contain the same concentrations of nutrients; also, each spice contains unique bioactive components that have varying effects on microbial growth (Liu et al., 2017).

### LAB Survival in a Simulated Upper Gastrointestinal Tract Is Significantly Improved by the Addition of Ginger and Cayenne to Tibicos

Though spices have been found to affect fermentation and storage, they may also have significant protective effects during digestion. The mechanisms are likely related to their antioxidant, antimicrobial, and prebiotic properties. A 2019 human randomized placebo-controlled pilot study by Lu et al. (2019) showed that consumption of a spice mixture, which included ginger and cayenne, resulted in significant modification of gut microbiota due to spices’ prebiotic effects. In our study, the addition of cayenne and ginger significantly improved LAB counts (*p*<0.05) compared to plain tibicos, while the viability of yeasts was not affected (Figure 4). However, cayenne LAB survival rates (Table 2) were significantly higher at 82% (*p*<0.05) than ginger (77%), plain (74%), and turmeric (73%). Cayenne and plain tibicos had (80%) significantly higher (*p*<0.05) yeast survival rates than ginger (78%) and turmeric (77%). Antioxidant bioavailability and activity is increased by the gastrointestinal environment, as shown in simulated gastric and duodenal digestion studies of various fruits and vegetables (Pérez-Vicente et al., 2002; Bouayed et al., 2011). Valero-Cases et al. (2017) attributed high microbial survival in fermented pomegranate juice to the increased bio-accessibility of antioxidant phenolic compounds before *in vitro* digestion, enhancing the ability of LAB to survive *in vitro* digestion. The prebiotic effects and high antioxidant capacity of spices such as ginger and cayenne may synergistically improve tibicos microbial survival in a simulated digestive environment. Our study suggests that microbes in tibicos can withstand digestive fluids and low pH, and that added spices may improve microbial viability.

**Table 2:** The addition of cayenne powder to tibicos significantly increases lactic acid bacteria (LAB) survival rate during *in vitro* digestion.

Treatments	LAB survival rate (%)	Yeast survival rate (%)
Plain tibicos	74.5±2.6^ac^	80.2±0.7^ac^
Ginger tibicos	77.0±0.7^a^	77.8±0.6^bd^
Cayenne tibicos	81.6±2.0^b^	80.3±0.2^a^
Turmeric tibicos	73.2±2.1^c^	77.3±1.1^de^

*Percentage survival of tibicos-associated LAB after simulated gastric and small intestinal digestion. Means within a column with different superscripts (a–c) are significantly different (p<0.05). The results expressed as mean (n=3)±SD (standard deviation)*.

### When Digested as Part of Naturally Produced Whole Fermented Foods, LAB and Yeasts Have High Survival Rates

Probiotic microorganisms must be able to endure the adversity of gastrointestinal travel, from salivary enzymes, to gastric acids, bile acids, and pancreatic juices (Food and Agriculture Organization of the United Nations and World Health Organisation, 2002; Derrien and van Hylckama Vlieg, 2015). Being naturally protected by their coevolved food matrices may assist in their effective transport to the large intestine. To our knowledge, the present study is the first to apply sauerkraut and tibicos in their whole food form to a simulated digestion model. Our aim was to investigate whether microbes in fermented foods were better able to survive the digestive tract when protected by their established food matrices.

In all four sauerkraut treatments, LAB counts fell significantly after *in vitro* digestion (Figure 3; *p*<0.05). Salt concentration and use of starter cultures did not affect LAB survival after simulated digestion. LAB counts of all sauerkraut treatments experienced an approximate 2 log loss. Overall, approximately 6 out of 8 log cfu/ml of LAB survived GI simulation, with a microbial survival rate of around 72% (Table 1). This two-log unit loss of LAB concurred with whole food *in vitro* digestion studies of other fermented foods; for example, LAB-fermented milks (Faye et al., 2012) and fermented vegetable juices (Değirmencioğlu et al., 2016). These microbial survival rates may be ascribed to low sensitivity to acidic pH, high hydrophobicity, and low sensitivity to bile salts (Zanirati et al., 2015). In contrast, Beganović et al. (2014) observed that 25 LAB strains isolated from sauerkraut and then applied as a pure culture without the food matrix underwent a 3–5 log loss (from 9 log cfu/ml to between 4 and 6 log cfu/ml) post-*in vitro* digestion. This indicates that LAB delivered in a sauerkraut whole food matrix may be further protected from the GI environment by their natural incorporation with suitable food substrates. As such, sauerkraut may be an appropriate matrix for the delivery of probiotic strains, whether inoculated or spontaneously fermented.

Our study of tibicos showed significant microbial losses after simulated digestion (*p*<0.05; Figure 4), with a one to two log unit loss of LAB and yeast after the small intestinal phase. The LAB survival rate was around 73–82%, while yeast survival was around 77–80% (Table 2). Değirmencioğlu et al. (2016) conducted an *in vitro* digestion study with fermented pomegranate juice containing LAB and two *Saccharomyces* yeasts commonly found in tibicos. They found that, depending on the yeast involved, LAB survival rates were between 67 and 73% (*S. cerevisiae*) and 74 and 77% (*S. boulardii*); our study had similar findings. However, when it comes to yeast survival, our rates were lower than those found in Değirmencioğlu et al.’s study (92–98%). This indicates that LAB and yeast survive digestion in whole food matrices. In regard to *in vitro* survival of strains isolated from tibicos, Romero-Luna et al. (2020) found that *L. casei* CT12 extracted from tibicos grains had a 40% survival rate after *in vitro* digestion (2020); another of their studies (2019) found that *S. cerevisiae* C41 isolated from tibicos had a 78% survival rate post-*in vitro* digestion. A 2019 study by Angelescu et al. (2019), *L. plantarum* CR1 isolated from tibicos had a survival rate of 79% after *in vitro* digestion. These varying results may be due to heterogeneous methodologies. The needs of microbes are strain specific, and while some may benefit from integration into a food matrix-based microbial community, others may not. Sequencing of the microbial communities throughout fermentation, storage and simulated digestion would help ascertain which species of LAB and yeasts may benefit from being integrated in a whole food matrix.

### Whole Digested Tibicos and Sauerkraut Change Composition and Relative Abundance of Bacteria During Colonic Fermentation

Despite the rapid increase in studies focused on the interaction of isolated fermented food components and microorganisms with the gut microbiome, these complex interactions are still poorly understood (Douillard and de Vos, 2019).

In our study, bacterial relative abundance and composition in the control and digested fermented food samples were affected by *in vitro* colonic fermentation in a similar way, due to the resident colonic microbiota in the feces (Aguirre and Venema, 2017). *Firmicutes*, *Fusobacteria*, and *Proteobacteria* were the dominant phyla in all samples (Figures 5, 6). However, there were some marked differences in relative abundance between the control and the fermented food samples. *Bacteroidetes* abundance dropped in tibicos and sauerkraut samples, while *Firmicutes* abundance increased or remained at a similar level to the control (Figures 5A, 6A). This finding was similar to other *in vitro* studies of the impact of probiotic goat milk with passionfruit by-product (Casarotti et al., 2020) and snow chrysanthemum polysaccharides (Wu et al., 2021b) on gut microbiota. *In vivo* studies in mice with LAB-fermented goat milk (Chen et al., 2020) and kefir (Hsu et al., 2018) also observed an increased *Firmicutes* to *Bacteroidetes* (F/B) ratio in the treatment group. On the other hand, other polysaccharides, from okra (Wu et al., 2021c), flaxseed (Zhou et al., 2020), and mushrooms (Liu et al., 2021), observed increased *Bacteroidetes* abundance, and reduced *Firmicutes*, with a lower F/B ratio. Polyphenols act as prebiotics in the gut and have also been shown to influence F/B ratio through the inhibition of certain bacterial species (Loo et al., 2020). This indicates that changes in gut microbiota are dependent on the substrates involved. F/B ratio must be considered with the abundance of particular bacterial species, the interplay with microbial diversity and the complexity of individual contributing factors, e.g., host genetics, baseline microbiota, and comorbidities.

Compared to the blank and the control, colonic fermentation of all digested sauerkraut and tibicos samples led to an up to 85-fold increase in *Megasphaera*, a genus of Firmicutes bacteria (Figures 5C, 6C). *Megasphaera* sp. have been found to have important metabolic roles in the human and porcine gut microbiome: *M. eldensii*, *NM10* and *BL7* produce essential amino acids and vitamins in the gut (Shetty et al., 2013) and utilize ruminal lactate to produce the beneficial short-chain fatty acid, butyrate (Hashizume et al., 2003; Kim et al., 2011). Polysaccharides have been found to encourage *Megasphaera* growth during *in vitro* fecal fermentation (Wu et al., 2021a,b,c). In our study, it is likely that indigestible polysaccharides from tibicos and sauerkraut were available in sufficient quantities during colonic fermentation to encourage *Megasphaera* growth.

Salt concentration and inoculation of sauerkraut did not have an observable effect on relative abundance of gut microbiota (Figure 5). However, the unfermented cabbage with 1.5% salt treatment had differing relative abundances compared to the control and sauerkraut samples. Colonic fermentation of the unfermented cabbage led to an expansion of *Enterobacter* (0–38%) as the dominant genus, with lower abundances of *Escherichia*, *Fusobacterium*, and *Megasphaera* than the sauerkraut. *Lactobacillus* and *Bifidobacterium* relative abundance was lower than the control but were two times and 10 times higher, respectively, than the sauerkraut samples. During the early stages of sauerkraut fermentation, Enterobacteriaceae levels in the food matrix have been shown to remain high (Lavefve et al., 2021). Our study, concurrent with others, observed increasing LAB counts during fermentation, with a peak at around 13days. This suggests that the unfermented cabbage is undergoing an early fermentation process in the simulated colon, leading to domination by *Enterobacter* and rapid LAB growth. These findings suggest that the impact of fermentation and storage on microbial counts is one of the major reasons for differences in bacterial relative abundance at the gut level. As we did not measure fiber content throughout our study, it is difficult to assess whether this is simply due to the prebiotic effect of unfermented fiber from the cabbage. Other possible mechanisms driving these differences include the microbial transformation of substrates prior to consumption and ingested microbes, affecting the bioavailability of polysaccharides and phenolic compounds in the digestive tract.

Currently, studying microbial communities in whole fermented foods has its drawbacks, in that only specific isolated strains can be further assessed for other prerequisites of probiotic potential and safety. A better understanding of the interplay between substrates and microbes in whole fermented foods requires sequencing of microbes, and monitoring of phenolic compounds, polysaccharides and microbial metabolites (including short-chain fatty acids), during fermentation, storage, and *in vitro* digestion. Although the harmonized static *in vitro* digestive system we used is well accepted in the study of food (Lucas-González et al., 2018), it has its limitations as an *in vivo* substitute (Nissen et al., 2020). Complex, multi-omics dynamic GI digestion and fermentation models would allow deeper investigation of how fermented food components interact, and how they may elicit shifts in the diversity, abundance, and richness of core microbial communities over time (Nissen et al., 2020; Ji et al., 2021).

The present study showed that storage affects microbial survival in both low-salt sauerkraut and tibicos, with excessive storage having a slight but significant detrimental impact on LAB and yeast counts. This indicates that tibicos should be consumed within 28days, while sauerkraut should be stored no longer than 7weeks. LAB and yeasts in both foods are able to adequately survive the low pH environment of fermentation, with resulting microbial counts high enough to be considered probiotic. Ginger and cayenne were observed to significantly enhance LAB survival during fermentation, storage, and GI digestion, likely due to inherent prebiotics, such as fiber, and antioxidant capacity. Our *in vitro* digestion results suggest that both sauerkraut and tibicos contain acid, enzyme, and bile tolerant LAB and yeasts, some of which have better survival rates when consumed in a whole food matrix. Sauerkraut and tibicos LAB and yeasts were shown to shift composition and relative abundance of gut microbiota. In relation to their survival in the GI tract and effects on human health, dynamic *in vitro* models, human cell studies, and clinical trials are required.

## Data Availability Statement

The datasets presented in this study can be found in online repositories. The names of the repository/repositories and accession number(s) can be found at: https://www.ncbi.nlm.nih.gov/, PRJNA754823.

## Author Contributions

MC and KH conceived and designed the study. FY, YW, and DL carried out the laboratory experiments and analyzed the data with MC. MC wrote the manuscript, which was edited by KH. All authors contributed to the article and approved the submitted version.

## Funding

MC was supported by an Australian Government Research Training Program (RTP) Scholarship through the University of Melbourne, Australia.

## Conflict of Interest

The authors declare that the research was conducted in the absence of any commercial or financial relationships that could be construed as a potential conflict of interest.

## Publisher’s Note

All claims expressed in this article are solely those of the authors and do not necessarily represent those of their affiliated organizations, or those of the publisher, the editors and the reviewers. Any product that may be evaluated in this article, or claim that may be made by its manufacturer, is not guaranteed or endorsed by the publisher.

## Footnotes

^1^ www.megazyme.com
